# *EPHB6* mutation induces cell adhesion-mediated paclitaxel resistance via *EPHA2* and *CDH11* expression

**DOI:** 10.1038/s12276-019-0261-z

**Published:** 2019-06-03

**Authors:** Sarah Yoon, Ji-Hye Choi, Sung Joo Kim, Eun-Ju Lee, Masaud Shah, Sangdun Choi, Hyun Goo Woo

**Affiliations:** 1 Department of Physiology, Ajou University School of Medicine, Suwon, Republic of Korea; 2 Department of Biomedical Science, Graduate School, Ajou University, Suwon, Republic of Korea; 3 Department of Molecular Science and Technology, Ajou University, Suwon, Republic of Korea

**Keywords:** Cancer therapeutic resistance, Oncogenes

## Abstract

Mutations affect gene functions related to cancer behavior, including cell growth, metastasis, and drug responses. Genome-wide profiling of cancer mutations and drug responses has identified actionable targets that can be utilized for the management of cancer patients. Here, the recapitulation of pharmacogenomic data revealed that the mutation of *EPHB6* is associated with paclitaxel resistance in cancer cells. Experimental data confirmed that the *EPHB6* mutation induces paclitaxel resistance in various cancer types, including lung, skin, and liver cancers. *EPHB6* mutation-induced paclitaxel resistance was mediated by an interaction with EPHA2, which promotes c-Jun N-terminal kinase (JNK)-mediated *cadherin 11* (*CDH11*) expression. We demonstrated that *EPHB6*-mutated cells acquire cell adhesion-mediated drug resistance (CAM-DR) in association with *CDH11* expression and RhoA/focal adhesion kinase (FAK) activation. Targeted inhibition of *EPHA2* or *CDH11* reversed the acquired paclitaxel resistance, suggesting its potential clinical utility. The present results suggest that the *EPHB6* mutation and its downstream EPHA2/JNK/CDH11/RhoA/FAK signaling axis are novel diagnostic and therapeutic targets for overcoming paclitaxel resistance in cancer patients.

## Cancer: Gene mutation spurs drug resistance

Mutations in a gene linked to cancer growth also underpin resistance to a common chemotherapy drug. Hyun Goo Woo and colleagues from Ajou University in Suwon, South Korea, analyzed a large database of genetic information on more than 1,000 human cancer cell lines looking for mutated genes associated with resistance to certain drugs. The top association was between mutated forms of *EPHB6*, a gene previously linked to tumor invasiveness, and resistance to paclitaxel, a chemotherapy drug used to treat a range of different cancers. They experimentally validated the finding in lung, skin and liver cancer cell lines. The researchers also identified two proteins activated by mutant *EPHB6* that promote the cell adhesion responsible for paclitaxel resistance. Inhibiting these two proteins reversed the drug resistance in cells, suggesting the therapeutic value of targeting this pathway.

## Introduction

Recent advances in the large-scale profiling of pharmacogenomic data, such as the Cancer Cell Line Encyclopedia (CCLE), have led to the identification of associations between genomic aberrations and drug sensitivity in cancer. Mutations in cancers are therefore considered diagnostic and therapeutic targets for the management of cancer patients. For example, targeting mutations in *EGFR* or *BRAF* in cancer is a strategy for patient-specific precision management in the clinic. In a previous work by our group, the recapitulation of CCLE data indicated that mutation of *sulfatase-2* increases sorafenib sensitivity in liver cancer patients^1^, suggesting that pharmacogenomic data are useful resources for identifying novel diagnostic and/or therapeutic targets. Here, we reanalyzed CCLE data to identify novel targetable mutations related to the acquisition of drug resistance. The results indicated that the *ephrin type-B receptor 6* (*EPHB6*) mutation may induce paclitaxel resistance.

The ephrin receptor (EPH receptor) subfamily is the largest subfamily of receptor tyrosine kinases, comprising 14 members in vertebrates, namely, ephrin type-A (EPHA) receptors 1–10 (*EPHA1–A8* and *EPHA10*) and ephrin type-B (EPHB) receptors 1–6 (*EPHB1–B4* and *EPHB6*)^2,3^. EPH receptors and ephrins play critical roles in various biological functions, such as embryonic patterning, nervous system development, and angiogenesis. However, the deregulated activation of ephrin/EPH receptor signaling in humans leads to tumor development and/or progression. The overexpression of the EPH receptor and ephrins has been shown in various cancer types. The upregulation of EPH receptors and ephrins is associated with poor prognosis and high vascularity in cancer, suggesting its detrimental effect on tumor progression. Unlike other EPH receptors, *EPHB6* lacks tyrosine kinase activity^4,5^ and shows tumor-suppressive effects^6–8^. A recent study showed that *EPHB6* expression is associated with better recurrence-free survival and increased drug sensitivity in triple negative breast cancers^9^. However, although recurrent mutations in *EPHB6* are observed in various types of cancer, the effect of *EPHB6* mutations on drug resistance remains to be investigated.

The present study investigated the effect of the *EPHB6* mutation on paclitaxel resistance in various cancer types. The results showed that the *EPHB6* mutation leads to the acquisition of cell adhesion-mediated drug resistance (CAM-DR) through a mechanism involving ephrin type-A receptor 2 (*EPHA2*) and *cadherin 11* (*CDH11*) expression. The present results suggest a novel mechanism underlying paclitaxel resistance in cancer patients and identify EPHB6 as a novel therapeutic target and/or biomarker for paclitaxel resistance in cancer patients.

## Materials and methods

### Pharmacogenomic data analysis

Mutations and drug sensitivity data from CCLE were analyzed. In brief, mutational features were categorized as described previously^10^. The mutation features of damaging loss-of-function (LOF) mutations, including nonsense, frameshift indel, and splice sites, were classified as “mutLOF”. The missense mutations were classified as “nnMS”. Combined mutation features of mutLOF and nnMS were classified as “mutLOF_nnMS”. The association of drug response with gene mutations was evaluated by applying Fisher’s exact test and the regularized elastic net regression analysis, and novel candidate drug-mutation pairs were selected by applying a prior knowledge-based filtering method, as described previously^1^ (for details see Supplementary Methods).

### Gene expression constructs and lentiviral vector transfection

Lentiviral constructs expressing *CDH11* shRNA and *JUN* shRNA were purchased from Sigma-Aldrich (St. Louis, MO, USA). The *EPHB6*-wild type, *EPHB6*-Q926R, *EPHB6*-del915-917 cDNA constructs were cloned into pCDH-CMV-MCS-EF1-Puro, a lentiviral vector for cDNA expression (System Biosciences, Mountain View, CA, USA). All lentiviral vectors were transfected into 293TN cells (System Biosciences) with Lipofectamine 3000 transfection reagent (Invitrogen, Waltham, MA, USA). Particles were collected 2 days after the transfection of the lentiviral plasmids and used to infect cancer cells. Lentivirus-infected cancer cells were puromycin-selected for 1 week.

### RNA-seq profiling

Total RNA was extracted from each sample using the mirVana Total RNA Extraction Kit (Ambion, Austin, TX). The sequencing library for RNA was constructed using the TruSeq RNA Sample Preparation Kit (Illumina, San Diego, CA) according to the manufacturer’s instructions. The sequencing reaction was performed on an Illumina NextSeq 500 for paired end reads (2 × 75 bp) with coverage greater than 30 million reads per sample. The raw image data were transformed and stored in the FASTQ format. The sequence reads were mapped to the human reference genome (hg38), and RNA abundance was estimated by using Tophat and Cufflinks with default parameters, and log2 transformed FPKM (fragment per kilobase of transcript per million mapped reads) values were used.

### In vivo experiments

Vector, WT (wild type), or Q926R cells (1 × 10^7^ cells/100 µl) and Matrigel (Corning, Bedford, MA, USA) 100 µl mixtures (total, 200 µl/head) were injected subcutaneously in the right rear dorsal flank region of Balb/c nude mice. When the tumor volume reached ~50 mm^3^, the mice were randomized into two treatment groups: control, 20 mg/kg paclitaxel. Paclitaxel was administered on days 1, 3, and 5 via intraperitoneal injection^11^. The tumors were measured using an optical caliper with a 3-day interval, and the tumor size was calculated using the following formula: length × (width)^2^ × 0.5. All surgical and experimental procedures were approved by the institutional animal care and use committee at Ajou University, College of Medicine.

### RhoA GTPase activity assay

RhoA activity was measured by using a kit from Cell Biolabs (San Diego, CA, USA). Briefly, the cell lysates were incubated with agarose beads coupled to the Rho-binding domain (RBD) of Rhotekin. The amount of bound RhoA was measured by western blot analysis using an anti-RhoA antibody.

### Cell adhesion assay

Cell adhesion was measured by a colorimetric-based assay (CytoSelect 48-Well Cell Adhesion Assay; Cell Biolabs Inc.) according to the manufacturer's instructions. Briefly, the cells were serum starved for 24 h prior to seeding onto collagen type IV-coated adhesion plates at a concentration of 1 × 10^6^ cells/ml in serum-free media. The cells were incubated for 90 min. Non-adherent cells were gently removed by several washes with 1× PBS, then the adherent cells were fixed with 3.7% formaldehyde and stained with Coomassie Brilliant Blue. The adherent cells were dissolved in an extraction solution, and the absorbance of this solution was measured at 560 nm in a microplate reader.

### In vitro drug sensitivity assay

To estimate CAM-DR, an in vitro drug sensitivity assay was performed in six-well plates as previously described^12^. Cells (1 × 10^3^ cells) were preincubated with or without the indicated drugs for 15 min and then adhered to plates coated with collagen type IV. After overnight incubation at 37 °C for adhesion, paclitaxel (10 nM) was added, and the incubation was continued for 24 h. After washing the plates twice with serum-free RPMI-1640, the cells were grown in complete culture medium for 14 days. The resulting colonies grown on the plates were stained with Coomassie Brilliant Blue, and the visible number of colonies was counted.

### Cell culture and other molecular experiments

The cells, antibodies, reagents, and the detailed methods for the molecular experiments of real-time PCR, immunoprecipitation, western blotting, confocal imaging, and cell proliferation, migration and, invasion assays are described in Supplementary Methods.

## Results

### *EPHB6* mutation increases paclitaxel resistance in cancer cells

**Fig. 1:**
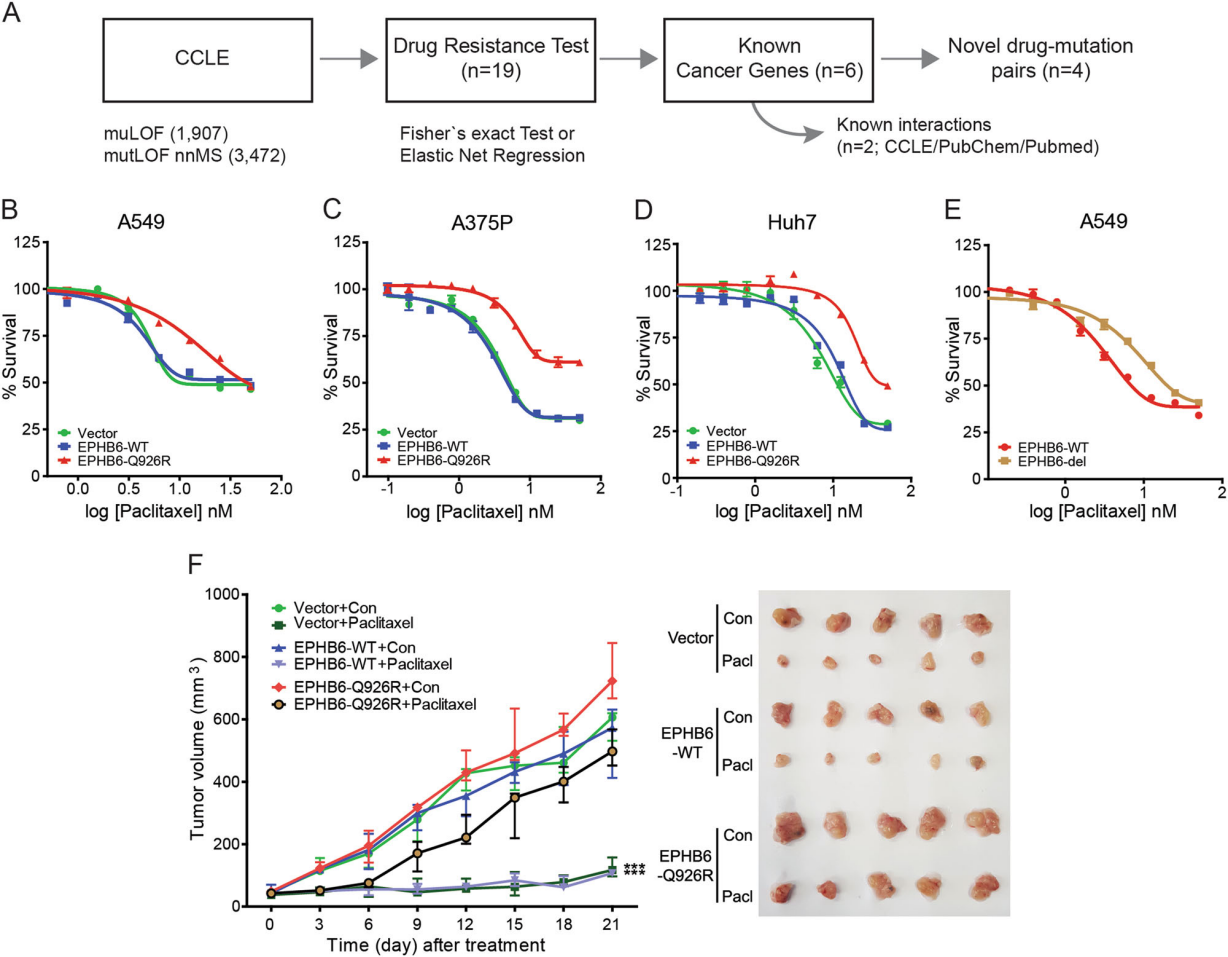
*EPHB6* mutation increases paclitaxel resistance in cancer cells. **a** A workflow for CCLE data analysis with prior knowledge-based filtering methods is shown. **b–e** Vector, *EPHB6* (WT), or *EPHB6* (Q926R)-overexpressing cells of A549 (**b**), A375P (**c**), HuH7 (**d**), and EPHB6 (WT) or *EPHB6* (del915-917)-expressing A549 cells (**e**) were treated with various concentrations of paclitaxel (0.2–50 nM) in 5% FBS-containing medium. After 72 h, IC_50_ values for paclitaxel were measured by WST-1 assays. **f** Male nude mice with Vector, WT, or Q926R cells were stratified into two groups (*n* = 5 for each group) and treated as described in the “Materials and methods”. Statistical significance is indicated (***P < 0.001, left). Pictures of the tumors resected from mice are shown (right)

The CCLE data were analyzed following a prior knowledge-based pipeline to detect novel mutation-induced alterations in drug resistance (for details see “Materials and methods” and Supplementary Methods). The analysis predicted four candidate gene mutation-drug pairs associated with drug resistance (Fig. 1a). Of these, *EPHB6* mutation-paclitaxel was the top ranked pair for the acquisition of drug resistance (Supplementary Table 1). Mutations in *EPHB6* were frequently found in lung cancers (6.5%) and melanomas (6.7%) (Supplementary Fig. 1), showing an association with a prometastatic phenotype^13^. Of the *EPHB6* mutations, nonsense mutations and a missense mutation, Q926R, showed the highest resistance to paclitaxel treatment (Supplementary Table 2). Therefore, we constructed *EPHB6*-Q926R mutant (Q926R)- and WT-expressing cells using an A549 lung cancer cell line (Supplementary Fig. 2A). We observed that the IC_50_ value for paclitaxel was markedly higher in Q926R cells (7.864 nM) than that in the Vector or WT cells (IC_50_ for Vector, 4.346 nM; IC_50_ for WT, 4.661 nM, Fig. 1b). We also observed *EPHB6* mutation-induced paclitaxel resistance in A375P melanoma and Huh7 liver cancer cells (Supplementary Fig. 2B, C and Fig. 1c, d), which may indicate *EPHB6* (Q926R) mutation-induced paclitaxel resistance in diverse cancer types.

The Q926R mutation was not observed in human cancer tissues from TCGA data; therefore, we decided to examine other EPHB6 mutations that were observed in human cancers. The Q926R mutation resides in the region of the EPHB6 protein between the tyrosine kinase catalytic domain (Tyrkc 655-900) and the sterile alpha motif (SAM 930-982). In this region, del915-917, D915G, and G914V were recurrently observed in human cancer patients^13,14^. Among these mutations, an in-frame deletion at 915-917 has been shown to increase the metastatic potential of lung cancers, implying its pathobiological significance^13^. We therefore evaluated whether the del915-917 mutation is associated with paclitaxel resistance. The *EPHB6*-del915-917 (del915-917) cells (Supplementary Fig. 2D), compared to the WT cells, exhibited increased IC_50_ values for paclitaxel (del915-917, IC_50_ = 7.52 nM; WT, IC_50_ = 4.661 nM) (Fig. 1e). These results indicate that *EPHB6* mutations, at least in this region (amino acids 901-929), lead to the acquisition of paclitaxel resistance.

In an in vivo xenograft mouse model, paclitaxel treatment significantly reduced tumor volume in Vector and WT tumors, whereas it had no effect on Q926R tumors (Fig. 1f). Taken together, these results suggest that the mutation of *EPHB6* induces paclitaxel resistance in tumor cells.

### *EPHB6* (Q926R) interferes with EPHA2 degradation by c-Cbl

**Fig. 2:**
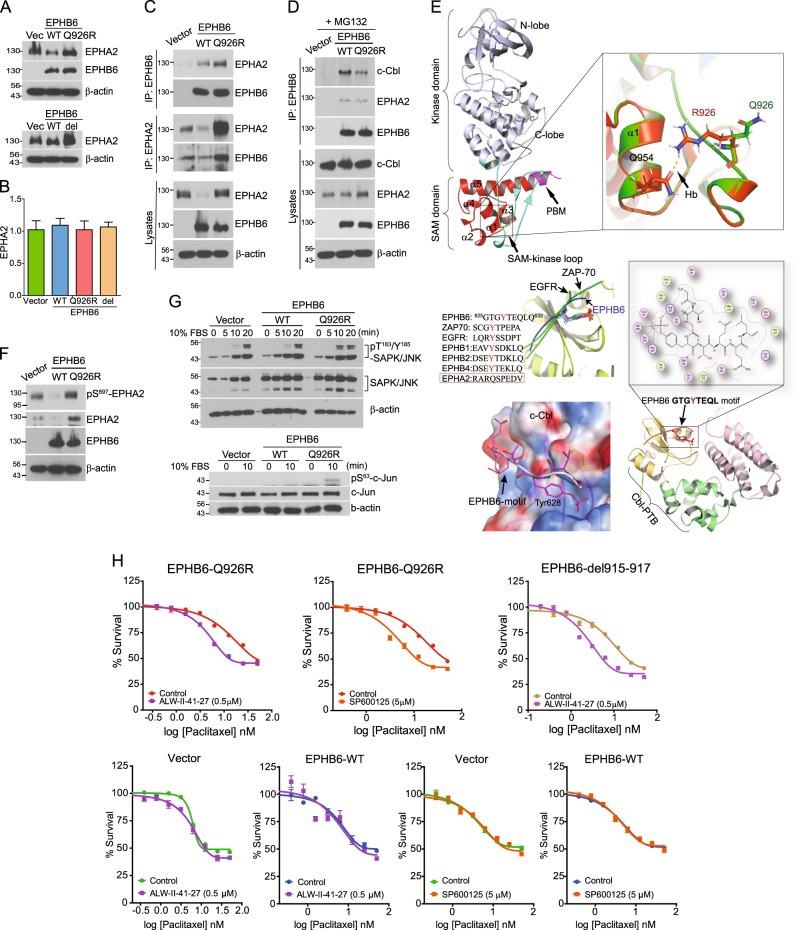
*EPHB6* (Q926R) interferes with EPHA2 degradation by c-Cbl. **a** Vector, WT, Q926R, or del915-917 cells were subjected to western blotting with the indicated antibodies. **b** *EPHA2* mRNA expression levels were measured by qRT-PCR in the indicated cells. **c** Cell lysates are immunoprecipitated with anti-EPHB6 or anti-EPHA2 antibodies. The amount of the pulled-down proteins was measured by western blot analysis. The expression levels of EPHB6 or EPHA2 in total cell lysates were used as input controls. **d** The indicated cells were treated with MG132 (10 µM) for 3 h, and their cell lysates were immunoprecipitated with anti-EPHB6 antibody, followed by western blot analysis. **e** The kinase (gray) and SAM (red) domains are connected by a flexible loop (light green). The c-terminal portion of the SAM domain contains a PDZ-binding motif (pink). The EPHB6-WT (green) and EPHB6-Q926R mutant (orange) are superposed, and the mutated residue is shown (stick). The mutant R926 interacts with Q954 in the α1 helix of the SAM domain, abrogating conformational alterations. The binding interface of the EPHB6 phosphotyrosine-containing motif and TKB domain of c-Cbl are shown (bottom). The phosphate moiety of the phosphotyrosine shows strong electrostatic interactions with the polar and positively charged residues (blue surface in electrostatic map). **f** Vector, WT, or Q926R cells were subjected to western blotting with the indicated antibodies. **g** The indicated cells are serum starved overnight and treated with 10% FBS for the indicated time periods, followed by western blot analyses with the indicated antibodies. **h** The indicated cells were treated with paclitaxel (0.2–50 nM) with or without ALW-II-41-27 (0.5 µM) or SP600125 (5 µM) in 5% FBS-containing medium. After 72 h, the IC_50_ values for paclitaxel are measured by WST-1 assays

EPHB6 interacts with several EPH receptors, such as EPHA2, EPHB2, and EPHB4^15,16^. In particular, *EPHA2* is frequently expressed in nonsmall cell lung cancers (90%) and metastatic melanomas (67%) in association with poor prognostic outcomes^17^. Based on this concern, we next investigated whether the interaction of EPHA2 with EPHB6 plays a role in the acquisition of paclitaxel resistance. EPHA2 was expressed at lower levels in WT cells than in Vector cells, whereas EPHA2 expression was higher in Q926R and del915-917 cells (Fig. 2a). These results strongly suggest that EPHA2 expression is involved in the acquisition of paclitaxel resistance associated with the EPHB6 mutation.

Unlike the protein expression levels*, EPHA2* mRNA expression levels did not differ significantly between Vector, WT, Q926R, and del915-917 cells (Fig. 2b), suggesting that the *EPHB6* mutation affects EPHA2 expression at the posttranscriptional level. Because EPHB6 interacts with EPHA2 and suppresses oncogenic signaling^18^, we examined whether mutations in EPHB6 affected its interaction with EPHA2. The results showed that the amount of EPHB6 (WT) co-immunoprecipitated with EPHA2 was markedly diminished, whereas the interaction between EPHB6 (Q926R) and EPHA2 was markedly increased (Fig. 2c). This result suggests that the interaction of EPHA2 with EPHB6 affects the stability of EPHA2 protein at the posttranscriptional level. The stability of the EPHA2 protein is regulated by the c-Cbl ubiquitin ligase^19^; therefore, we evaluated the effect of c-Cbl on the stability of the EPHB6-EPHA2 complex. In the presence of the proteasome inhibitor MG-132 (10 µM), the amount of c-Cbl recruited to the EPHA2-EPHB6 (Q926R) complex was lower than that interacting with the EPHA2-EPHB6 (WT) complex (Fig. 2d). These findings indicate that the mutation of EPHB6 inhibits the recruitment of c-Cbl to the EPHA2-EPHB6 complex, suppressing the c-Cbl-induced degradation of EPHA2.

To further support our finding, we analyzed structural alterations in the *EPHB6* mutant and its interaction with c-Cbl. The conformational rearrangement of the SAM domain of EPHB6 may affect the flexibility and the optimum length of the loop between the SAM and kinase domains. Indeed, we observed that the arginine residue of the Q926R mutant was spatially close to the α1 helix-containing polar glutamine 954 and serine 958 (Fig. 2e, top). This topology may compromise the flexibility of the loop by facilitating new contacts with the nearby glutamine in the SAM domain. In addition, receptor tyrosine kinases contain a consensus motif (D/N)XpYXX(D/E0φ), which is recognized by Src homology 2 (SH2) or the tyrosine kinase binding (TKB) domain of c-Cbl^20^. We observed that EPHB6, but not EPHA2, had a similar phosphotyrosine-containing motif in the juxtamembrane region, which may recruit c-Cbl by establishing contacts with the TKB domain of c-Cbl (Fig. 2e, bottom). Thus, we suggest that the structural alteration of the *EPHB6* mutation reduces the flexibility of the SAM domain, suppressing c-Cbl recruitment, which in turn suppresses the degradation of EPHA2 by c-Cbl.

Next, we investigated the downstream signaling pathways of EPHA2. The tumor-promoting effects of *EPHA2* are mediated by ligand-independent signaling involving serine S897 phosphorylation^21,22^. Consistently, the present results showed that EPHA2 phosphorylation at S897 was lower in WT cells and significantly higher in Q926R cells than in Vector cells (Fig. 2f). This finding may indicate that ligand-independent EPHA2 signaling is activated by the Q926R mutation but suppressed in WT cells.

Because *c-Jun N-terminal kinase* (*JNK*) is a downstream gene in the EPHA2 ligand-independent signaling pathway that promotes the aggressive behavior of cancer cells, we examined JNK activation status in our model^22^. The active forms of JNK and c-Jun were increased significantly at 10 min after serum stimulation in Q926R cells but not in Vector and WT cells (Fig. 2g). Exposure of cells to the EPHA2 inhibitor ALW-II-41-27 (0.5 µM) or the JNK inhibitor SP600125 (5 µM) increased paclitaxel sensitivity in Q926R cells, but not in Vector and WT cells (Fig. 2h). These results indicate that EPHA2/JNK is involved in the paclitaxel resistance induced by the EPHB6 mutation. Taken together, these results suggest that the EPHB6 mutation promotes ligand-independent EPHA2 signaling and JNK activation.

### *CDH11* is a downstream effector gene for *EPHB6* (Q926R)-induced paclitaxel resistance

**Fig. 3:**
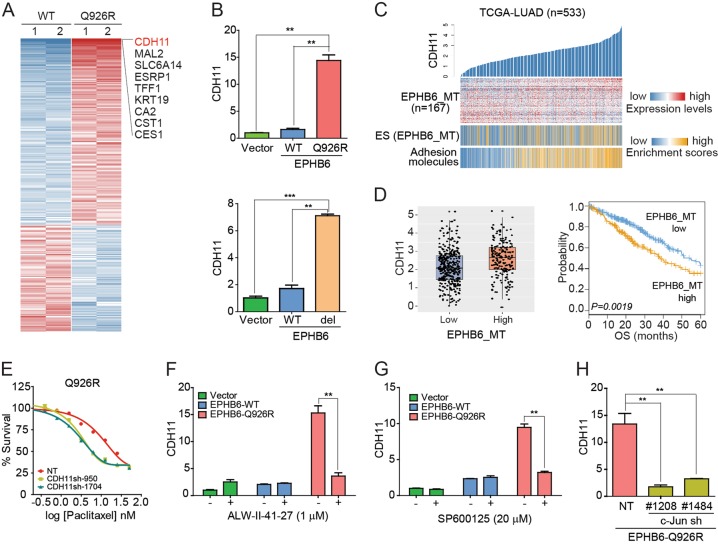
*CDH11* is a downstream effector gene for *EPHB6* (Q926R)-induced paclitaxel resistance. **a** A heatmap shows the differentially expressed genes between WT and Q926R cells. **b** *CDH11* mRNA expression levels in the indicated cells were measured by qRT-PCR. **c** Expression of *CDH11* (top), *EPHB6*_MT signature (*n* = 167, middle), and adhesion-related gene signature (*n* = 1032, bottom) are shown in lung adenocarcinoma data from TCGA (TCGA-LUAD, *n* = 533). The enrichment scores (ES) for gene signatures are calculated by a preranked GSEA method. The patients were stratified into two groups of high (*n* = 187) and low expression (*n* = 346) *EPHB6*_MT signatures based on their average value. **d** A boxplot showing the differential expression of *CDH11* between the patient groups (left). Kaplan–Meier plot analysis showing the overall survival between the patient groups (right). **e** Q926R cells transfected with nontargeting (NT) shRNA or *CDH11* shRNA (#950 or #1704) were treated with paclitaxel (0.2–50 nM), and the IC_50_ values for paclitaxel were measured by WST-1 assays. **f**, **g** Indicated cells are treated with or without ALW-II-41-27 (1 µM) **f** or SP600125 (20 µM) **g** for 48 h. *CDH11* expression levels were measured by qRT-PCR. Values are presented as the means ± SEM of three replicates. **h** *CDH11* expression levels are measured in the Q926R cells transfected with nontargeting (NT) shRNA, *c-Jun* shRNA (#1208 or #1484) by qRT-PCR. Values are means ± SEM of three replicates. **P < 0.01 and ***P < 0.001

To identify potential effector genes associated with the Q926R mutation, we performed RNA-seq profiling and identified genes differentially expressed in Q926R and WT cells (*i.e*., *EPHB6*_MT, *n* = 171, and *EPHB6*_WT, *n* = 98, fold difference >0.5, Fig. 3a and Supplementary Table 3). Gene ontology analysis revealed that compared to WT cells, Q926R cells were highly enriched with cell localization-related functions (enrichment scores = 2.7, Supplementary Table 4). Among the *EPHB6*_MT genes, *CDH11* showed the greatest difference in expression between Q926R and WT cells. The expression of *CDH11* was assessed by qRT-PCR in Q926R and del915-917 mutant cells (Fig. 3b). In addition, to determine whether the *EPHB6*_MT signature has functional and clinical significance, the gene expression profiles of lung adenocarcinoma cohorts (TCGA-LUAD, *n* = 533) were analyzed. The results showed that *CDH11* expression was significantly correlated with enrichment scores for the expression of cell adhesion genes (*r* = 0.63, *P* = 7.19 × 10^−61^, Fig. 3c). Moreover, the enrichment scores of the *EPHB6*_MT signature were highly correlated with *CDH11* expression levels. The stratification of patients into two groups according to the *EPHB6*_MT enrichment scores showed that *CDH11* expression was higher in the high *EPHB6*_MT group (*n* = 187) than that in the low *EPHB6*_MT group (*n* = 346) (permutated *T*-test *P* = 1.14 × 10^−6^, Fig. 3d, left). Kaplan–Meier analysis showed that the high *EPHB6*_MT group had worse overall survival than the low *EPHB6*_MT group (hazard ratio = 1.60, *P* = 1.91 × 10^−3^, Fig. 3d, right). These results indicate that the *EPHB6*_MT signature, including *CDH11*, may play regulatory roles in cancer progression.

After confirming the functional significance of the *EPHB6*_MT signature, we further investigated the functional roles of *CDH11* in paclitaxel resistance. The shRNA-mediated knockdown of *CDH11* significantly reduced *EPHB6* mutation-induced paclitaxel resistance, indicating that *CDH11* is a potential downstream effector for acquired drug resistance (Fig. 3e and Supplementary Fig. 3). We next investigated whether EPHA2 activation promoted *CDH11* expression. Treatment with the EPHA2 inhibitor ALW-II-41-27 (1 µM) suppressed *EPHB6* (Q926R)-induced *CDH11* expression (Fig. 3f). Treatment with the JNK inhibitor SP600125 (20 µM) or *c-Jun* shRNAs also significantly suppressed *EPHB6* (Q926R)-induced *CDH11* expression (Fig. 3g, h). Taken together, these results suggest that the *EPHB6* mutation induces *CDH11* expression, resulting in the acquisition of paclitaxel resistance, which is mediated by the activation of EPHA2 and JNK.

### *EPHB6* (Q926R)-induced *CDH11* expression activates RhoA and stress fiber formation

**Fig. 4:**
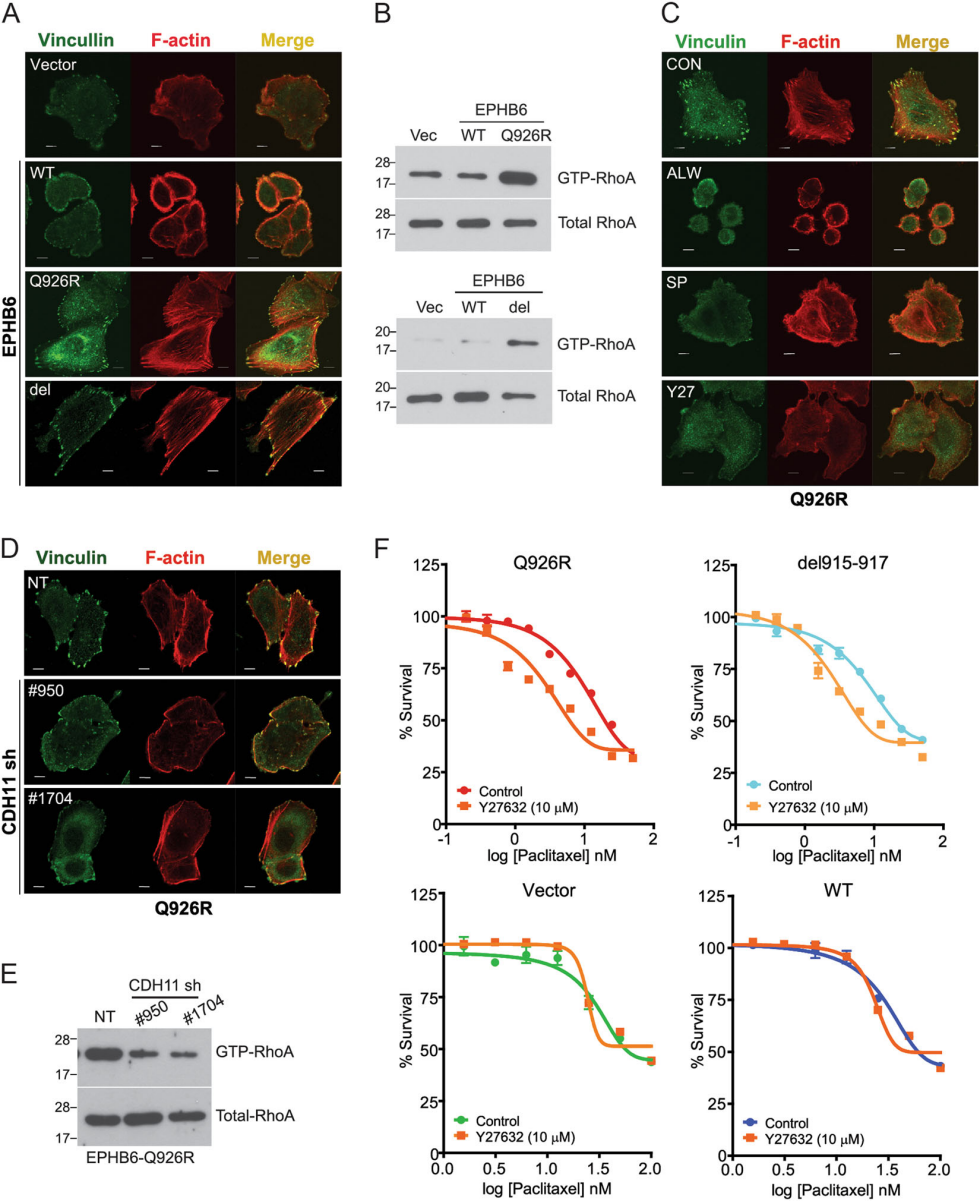
*EPHB6* (Q926R)-induced *CDH11* expression activates RhoA and stress fiber formation. **a** The indicated cells are fluorescently stained with anti-vinculin and Dylight 594 phalloidin. **b** Total cell lysates from the indicated cells are incubated with agarose beads coupled to the Rho-binding domain (RBD) of Rhotekin. The amount of the bound form RhoA and total RhoA were measured by western blotting with a RhoA antibody. **c** Q926R cells were treated with ALW-II-41-27 (1 µM), SP600125 (20 µM), or Y27632 (10 µM) for 24 h and then fluorescently stained with anti-vinculin and Dylight 594 phalloidin. **d**, **e** Q926R cells were transfected with NT shRNA or *CDH11* shRNA (#950 or #1704). Then, the cells were fluorescence-stained with anti-vinculin and Dylight 594 phalloidin (**d**), or the total cell lysates were incubated with agarose beads coupled to the Rho-binding domain (RBD) of Rhotekin. The amount of the bound form RhoA and total RhoA were measured by western blotting with a RhoA antibody (**e**). **f** Indicated cells were treated with paclitaxel (0.2–50 nM) with or without Y27632 (10 µM). IC_50_ values for paclitaxel were measured by WST-1 assays. The scale bar indicates 10 µm

*CDH11* is a cell adhesion molecule that activates the formation of cytoskeletal actin stress fibers, increasing the metastatic potential of cancer cells^23^. The present analysis showed that the production of stress fibers and focal adhesion molecules, such as vinculin, was higher in Q926R and del915-917 cells than that in Vector or WT cells (Fig. 4a). Because RhoA activation promotes stress fiber formation^24^, we performed a RhoA protein pull-down assay, which showed that GTP-bound RhoA protein levels were higher in Q926R and del915-917 cells than those in Vector or WT cells (Fig. 4b). In addition, treatment with EPHA2 inhibitor (ALW-II-41-27, 1 µM), JNK inhibitor (SP600125, 20 µM), or Rho-associated protein kinase inhibitor (Y27632, 10 µM) suppressed stress fiber and focal adhesion formation in Q926R cells (Fig. 4c). These results indicate that the *EPHB6* mutation induces stress fiber and focal adhesion formation and the EPHA2/JNK/RhoA pathway is involved in this process.

To determine whether *CDH11* is involved in the increased stress fiber and focal adhesion formation in *EPHB6* mutant cells, *CDH11* was knocked down (Supplementary Fig. 3), which suppressed stress fiber and focal adhesion formation as well as the expression of GTP-RhoA in Q926R cells but not in WT cells (Fig. 4d, e). Treatment with Y27632 (10 µM) rescued the acquired paclitaxel resistance in Q926R cells (Control, IC_50_ = 8.07 nM; Y27632, IC_50_ = 3.208 nM) and del915-917 cells (Control, IC_50_ = 7.52 nM; Y27632, IC_50_ = 3.928 nM) (Fig. 4f). Taken together, these results suggest that *EPHB6* mutation-induced *CDH11* expression promotes stress fiber and focal adhesion formation through the activation of EPHA2/JNK/RhoA signaling.

### CAM-DR is induced in Q926R cells

The effect of the *EPHB6* mutation on promoting stress fiber and focal adhesion formation implies that alterations in cell adhesion properties may play key roles in the acquisition of paclitaxel resistance. Indeed, CAM-DR is one of the mechanisms underlying the acquisition of drug resistance^25^. Moreover, a recent study showed that *CDH11* expression can promote cell adhesion^26^. We therefore evaluated the potential involvement of CAM-DR in *EPHB6* mutation-induced paclitaxel resistance.

**Fig. 5:**
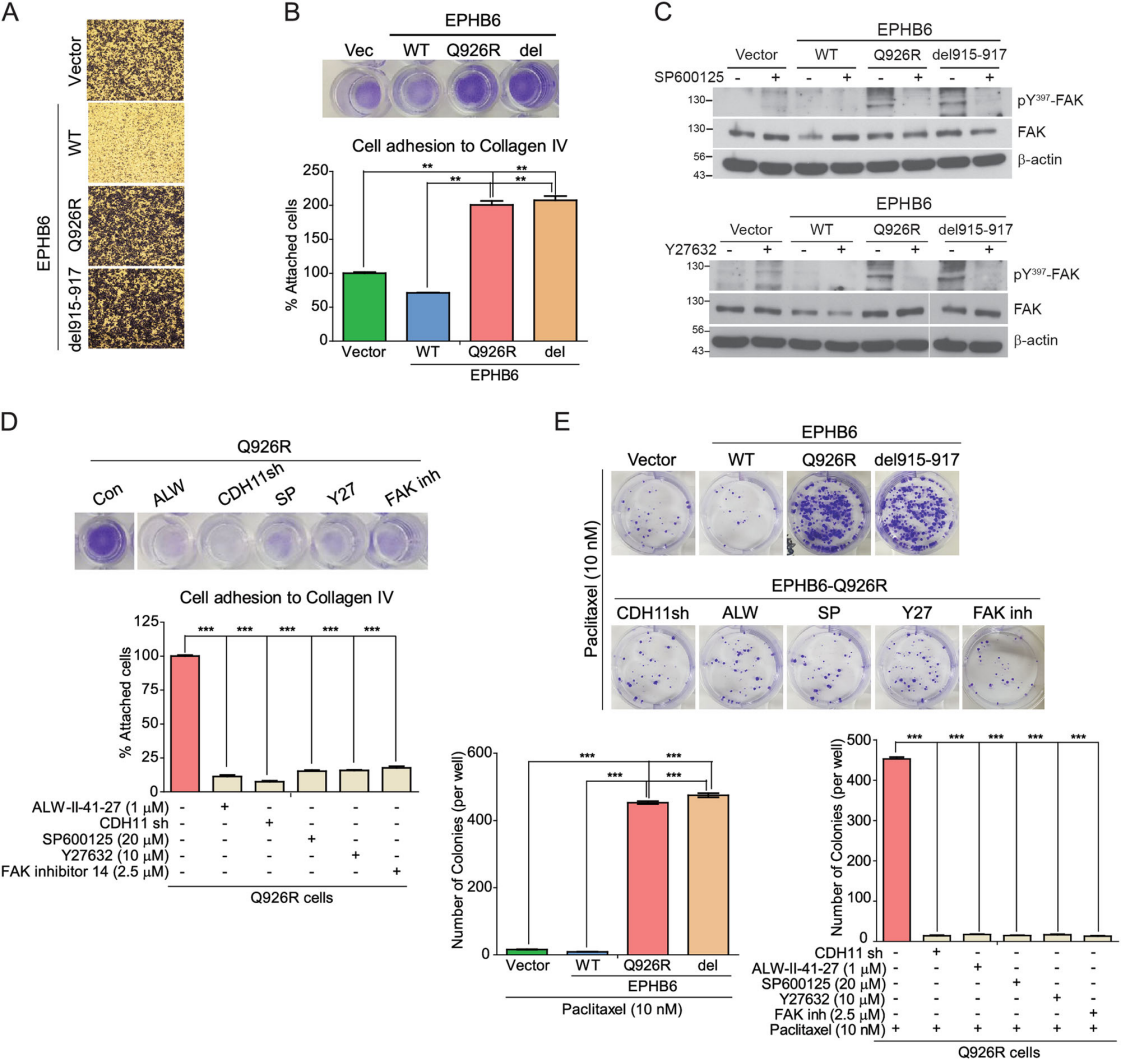
Cell adhesion-mediated drug resistance (CAM-DR) is induced in Q926R cells. **a** Cell invasion activities of the Vector, WT, Q926R, or del915-917 cells were measured by transwell invasion assays. After 4 h of incubation, the invaded cells were fixed and stained using light microscopy (magnification, ×200). **b** The indicated cells are attached to collagen type IV-coated 48-well plates for 1 h (100,000 cells/well). The adherent cells are stained (top), and the percentage of the attached cells is shown (bottom). **c** The indicated cells were treated with or without SP600125 (20 µM) (top) or Y27632 (10 µM) (bottom) for 48 h, respectively, followed by western blot analyses with the indicated antibodies **d** Q926R cells treated with or without ALW-II-41-27 (1 µM), *CDH11* shRNA (#950), SP600125 (20 µM), Y27632 (10 µM), or FAK inhibitor 14 (2.5 µM) were attached to collagen type IV-coated 48-well plates for 1 h (100,000 cells/well). The adherent cells were stained (top), and the percentage of the attached cells is shown (bottom). **e** The colony formation of the cells was measured after 14 days of incubation in a six-well plate coated with collagen type IV (top). The number of colonies formed from the indicated cells is counted (bottom). **P < 0.01 and ***P < 0.001

First, we evaluated the effect of *EPHB6* on cancer cell migration/invasion and growth. Previous studies have shown that *EPHB6* regulates cell motility and invasive potential rather than cell proliferation in different tumor types^7,27^. Similarly, we observed that compared with the Vector cells, the WT cells showed less migration/invasion, whereas no differences in proliferation were observed between the WT and Vector cells (Fig. 5a and Supplementary Fig. 4). However, Q926R cells showed a higher rate of proliferation and higher migration/invasion ability than did WT cells (Fig. 5a and Supplementary Fig. 4). In addition, the adhesion to collagen type IV (Col IV) was higher in Q926R and del915-917 cells than in Vector cells (Fig. 5b). Cell migration/invasion potential and adhesion ability were significantly lower in WT cells than in mutant cells, reflecting the tumor suppressor phenotype of these cells. These results suggest that the EPHB6 mutation confers CAM-DR, which is not observed in Vector or WT cells.

We next sought to identify molecular mediators of CAM-DR in *EPHB6* mutant cells. Focal adhesion kinase (FAK) is a key regulator of cancer cell invasion and cell adhesion. To determine whether *EPHB6* status regulates FAK activation, the phosphorylation of FAK at Y397 was examined. The results showed that FAK phosphorylation was increased in both Q926R and del915-917 cells, but not in Vector or WT cells (Fig. 5c). FAK regulates actin remodeling by activating JNK and RhoA^28^. In the present study, the inhibition of JNK (SP600125, 20 µM) or RhoA (Y27632, 10 µM) decreased FAK phosphorylation in mutant cells. In addition, increased adhesion in Q926R cells was abolished by exposure to ALW-II-41-27 (0.5 µM), *CDH11* shRNA, SP600125 (20 µM), Y27632 (10 µM), or FAK inhibitor (2.5 µM, Fig. 5d).

An in vitro drug sensitivity assay was performed to confirm the involvement of the *EPHB6* mutation in the acquisition of CAM-DR. For this purpose, the cells were adhered to Col IV-coated wells and treated with paclitaxel. Subsequently, colony formation was measured (for details, see “Materials and methods”). The results showed that colony formation after paclitaxel treatment was significantly higher in attached *EPHB6* mutant cells than in Vector or WT cells (Fig. 5e). These data indicate that *EPHB6* mutation-induced paclitaxel resistance is a CAM-DR process. Furthermore, CAM-DR induced by the *EPHB6* mutation was abolished by treatment with *CDH11* shRNA, ALW-II-41-27 (0.5 µM), SP600125 (20 µM), Y27632 (10 µM), or FAK inhibitor 14 (2.5 µM) (Fig. 5e). Taken together, these results suggest that the *EPHB6* mutation induces CAM-DR through the activation of EPHA2/JNK/CDH11/RhoA/FAK signaling.

## Discussion

**Fig. 6:**
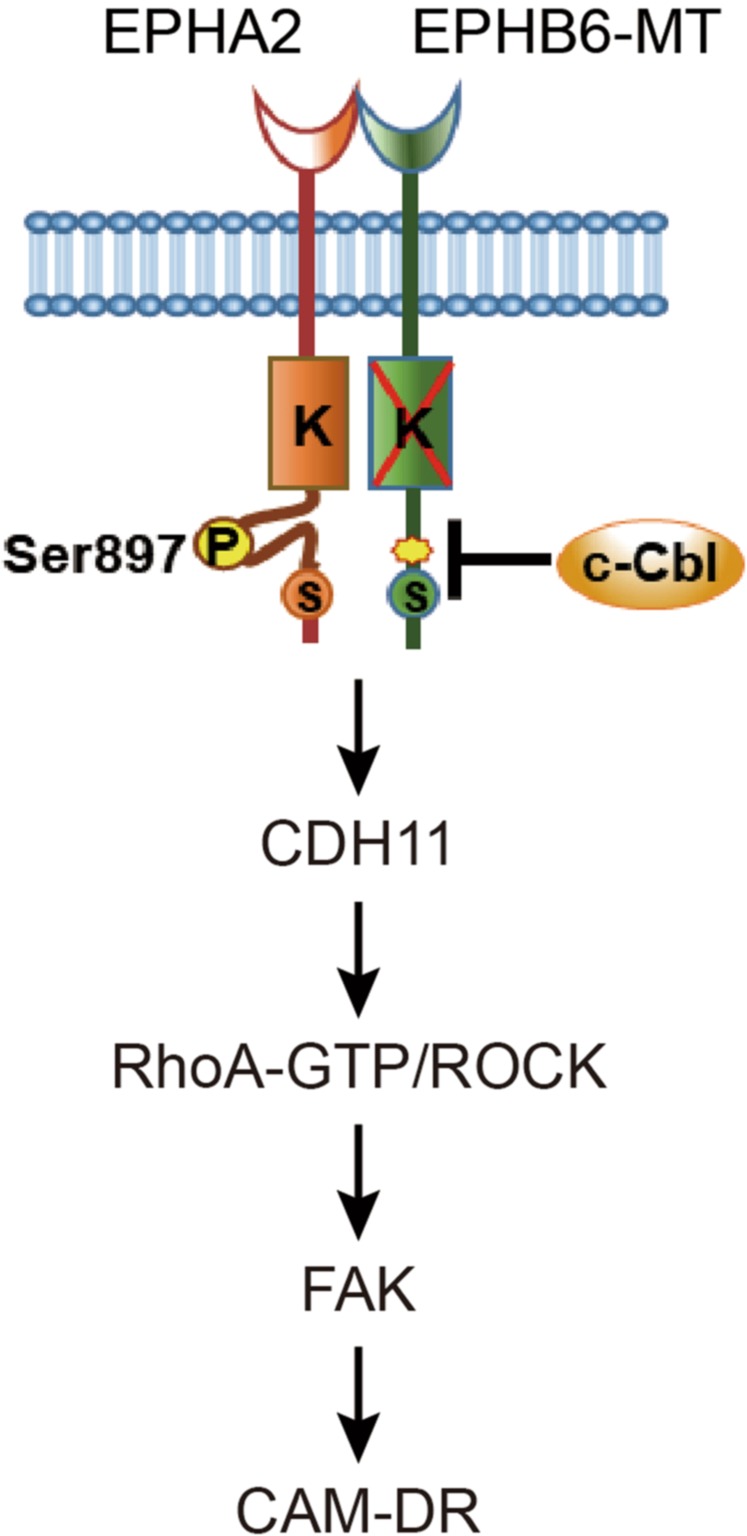
Graphical summary of the mechanisms for *EPHB6* mutation-induced paclitaxel resistance

In the present study, a previous knowledge-based CCLE data analysis predicted that the *EPHB6* mutation induces paclitaxel resistance in cancer cells, which was validated experimentally. We also demonstrated that the *EPHB6* mutation acquires CAM-DR. The *EPHB6* mutant interacted with EPHA2 and activated downstream JNK/CDH11/RhoA/FAK signaling. A graphical summary of our findings is shown in Fig. 6.

Although *EPHB6* lacks tyrosine kinase activity, its cytoplasmic domain is phosphorylated by EPHB1, EPHB4, or a Src family tyrosine kinase. EPHB6 interacts with EPHA2 and suppresses its oncogenic effect^18^. Consistent with the previous findings, we observed an oncosuppressive effect of EPHB6 (WT) mediated by an interaction with EPHA2 and the suppression of its oncogenic function (see Fig. 5). In contrast, the *EPHB6* mutant acquired a phenotype leading to the activation of EPHA2 and downstream JNK signaling (Fig. 2). This effect was mediated by the inhibition of c-Cbl recruitment, which in turn inhibited the degradation of EPHA2. The oncogenic function of EPHA2 is ligand-independent, as exogenous ephrin-A1 stimulation inhibits tumor cell proliferation^29^. Unlike the WT, mutant *EPHB6* induced *CDH11* expression and promoted paclitaxel resistance. Taken together with previous data, our findings suggest that the *EPHB6* mutation activates ligand-independent EPHA2 signaling, which modulates the different functional activities of the EPHB6 mutant and WT proteins.

*CDH11* is a mesenchymal cadherin that is frequently expressed in various cancer types in association with aggressive cancer behaviors, such as adhesion, migration, and metastasis^30,31^. Cell adhesion to the extracellular matrix is achieved by the activation of actin cytoskeleton remodeling and focal adhesion formation, specifically the assembly of actin into contractile stress fibers. Focal adhesions are dynamic complexes that contain proteins, such as integrins, FAK, paxillin, and vinculin. The binding of vinculin to F-actin is critical for cell-matrix adhesion^32^. The present data indicated that the expression of *CDH11* in *EPHB6* mutant cells leads to the acquisition of CAM-DR in association with increased stress fiber assembly and focal adhesion formation.

In conclusion, the present results suggest that the *EPHB6* mutation promotes cancer cell proliferation and migration/invasion and induces CAM-DR, and these effects are mediated by the stabilization of EPHA2 and the activation of downstream JNK/CDH11/RhoA/FAK signaling. The combined and precise targeting of these pathways might have therapeutic or diagnostic benefits in the management of paclitaxel resistance in cancer patients.

## Supplementary information

Supplementary Methods

Supplementary Figure

Supplementary Tables

## References

[1] Yoon S (2018). Recapitulation of pharmacogenomic data reveals that invalidation of SULF2 enhance sorafenib susceptibility in liver cancer. Oncogene.

[2] Menzel P, Valencia F, Godement P, Dodelet VC, Pasquale EB (2001). Ephrin-A6, a new ligand for EphA receptors in the developing visual system. Dev. Biol..

[3] Eph Nomenclature Committee (1997). Unified Nomenclature for Eph Family Receptors and Their Ligands, the Ephrins. Cell.

[4] Gurniak CB, Berg LJ (1996). A new member of the Eph family of receptors that lacks protein tyrosine kinase activity. Oncogene.

[5] Matsuoka H (1997). Expression of a kinase-defective Eph-like receptor in the normal human brain. Biochem. Biophys. Res. Commun..

[6] Ho DWH (2017). TSC1/2 mutations define a molecular subset of HCC with aggressive behaviour and treatment implication. Gut.

[7] Fox BP, Kandpal RP (2009). EphB6 receptor significantly alters invasiveness and other phenotypic characteristics of human breast carcinoma cells. Oncogene.

[8] Shen Q (2018). Barrier to autointegration factor 1, procollagen-lysine, 2-oxoglutarate 5-dioxygenase 3, and splicing factor 3b subunit 4 as early-stage cancer decision markers and drivers of hepatocellular carcinoma. Hepatology.

[9] Toosi BM (2018). EPHB6 augments both development and drug sensitivity of triple-negative breast cancer tumours. Oncogene.

[10] Barretina J (2012). The Cancer Cell Line Encyclopedia enables predictive modelling of anticancer drug sensitivity. Nature.

[11] Yuan H, Sun B, Gao F, Lan M (2016). Synergistic anticancer effects of andrographolide and paclitaxel against A549 NSCLC cells. Pharm. Biol..

[12] Sun L (2014). Gastric cancer cell adhesion to laminin enhances acquired chemotherapeutic drug resistance mediated by MGr1-Ag/37LRP. Oncol. Rep..

[13] Bulk E (2012). Mutations of the EPHB6 receptor tyrosine kinase induce a pro-metastatic phenotype in non-small cell lung cancer. PLoS ONE.

[14] Gylfe AE (2010). Somatic mutations and germline sequence variants in patients with familial colorectal cancer. Int J. Cancer.

[15] Fox BP, Kandpal RP (2011). A paradigm shift in EPH receptor interaction: biological relevance of EPHB6 interaction with EPHA2 and EPHB2 in breast carcinoma cell lines. Cancer Genom. Proteom..

[16] Kaenel P, Mosimann M, Andres AC (2012). The multifaceted roles of Eph/ephrin signaling in breast cancer. Cell Adhes. Migr..

[17] Brannan JM (2009). Expression of the receptor tyrosine kinase EphA2 is increased in smokers and predicts poor survival in non-small cell lung cancer. Clin. Cancer Res..

[18] Akada M, Harada K, Negishi M, Katoh H (2014). EphB6 promotes anoikis by modulating EphA2 signaling. Cell Signal.

[19] Walker-Daniels J, Riese DJ, Kinch MS (2002). c-Cbl-dependent EphA2 protein degradation is induced by ligand binding. Mol. Cancer Res.

[20] Peschard P, Ishiyama N, Lin T, Lipkowitz S, Park M (2004). A conserved DpYR motif in the juxtamembrane domain of the Met receptor family forms an atypical c-Cbl/Cbl-b tyrosine kinase binding domain binding site required for suppression of oncogenic activation. J. Biol. Chem..

[21] Jiang Y, Li Y, Zhu B (2015). T-cell exhaustion in the tumor microenvironment. Cell Death Dis..

[22] Song W, Ma Y, Wang J, Brantley-Sieders D, Chen J (2014). JNK signaling mediates EPHA2-dependent tumor cell proliferation, motility, and cancer stem cell-like properties in non-small cell lung cancer. Cancer Res..

[23] Row S, Liu Y, Alimperti S, Agarwal SK, Andreadis ST (2016). Cadherin-11 is a novel regulator of extracellular matrix synthesis and tissue mechanics. J. Cell Sci..

[24] Tojkander S, Gateva G, Lappalainen P (2012). Actin stress fibers-assembly, dynamics and biological roles. J. Cell Sci..

[25] Dickreuter E, Cordes N (2017). The cancer cell adhesion resistome: mechanisms, targeting and translational approaches. Biol. Chem..

[26] Langhe RP (2016). Cadherin-11 localizes to focal adhesions and promotes cell-substrate adhesion. Nat. Commun..

[27] Doi A (2009). Differential methylation of tissue- and cancer-specific CpG island shores distinguishes human induced pluripotent stem cells, embryonic stem cells and fibroblasts. Nat. Genet..

[28] Chen BH, Tzen JT, Bresnick AR, Chen HC (2002). Roles of Rho-associated kinase and myosin light chain kinase in morphological and migratory defects of focal adhesion kinase-null cells. J. Biol. Chem..

[29] Brannan JM (2009). EphA2 in the early pathogenesis and progression of non-small cell lung cancer. Cancer Prev. Res..

[30] Birtolo C (2017). Cadherin-11 Is a cell surface marker up-regulated in activated pancreatic stellate cells and is involved in pancreatic cancer cell migration. Am. J. Pathol..

[31] Zhu Q (2018). The role of cadherin-11 in microcystin-LR-induced migration and invasion in colorectal carcinoma cells. Oncol. Lett..

[32] Bays JL, DeMali KA (2017). Vinculin in cell-cell and cell-matrix adhesions. Cell Mol. Life Sci..

