# Supplementary material for: EPHB6 mutation induces cell adhesion-mediated paclitaxel resistance via EPHA2 and CDH11 expression
Source: Exp Mol Med. 2019 Jun 3;51(6):61. doi: 10.1038/s12276-019-0261-z (PMC6547695; doi:10.1038/s12276-019-0261-z)
Supplement: Supplementary file 1 — Supplementary Methods [file 12276_2019_261_MOESM1_ESM.docx]

## Supplementary Methods

### Pharmacogenomic data analysis

The cell lines were stratified into two groups of “resistant” (R) and “sensitive” (S) groups based on the median log-transformed IC_50_ values of each drug across all the cell lines. Then, the associations between the drug sensitivity groups and mutation profiles were estimated by applying two different methods of regularized elastic net regression analysis and Fishers’ exact T-test, respectively. In brief, regularized elastic net regression analysis was performed using R package library “glmnet” as following the procedures described previously (10). Parameter optimization for α and λ were estimated by 10 leave-group-out cross validations, then the significance of the drug-mutation interactions was determined by performing 200 bootstrap procedures. The mutation features with increased drug sensitivity and mutation frequency greater than 10 were considered in this analysis. Fisher’s exact test was also performed, and the significance was determined by odds ratios (OR) < 0.3 and *P*<0.01.

To identify mutant-specific alteration of drug resistance, we evaluated CCLE data as described in Materials and Methods. First, we selected seven tissue types that have data for more than 25 different cell lines. These included tumor tissue types from breast, central nervous system, hematopoietic and lymphoid tissue, lung, ovary, pancreas, and skin. In total, the selected data consisted of 290 cell lines and 33,444 sequence variants. The mutations were categorized according to the features of mutLOF (n=1,907) and mutLOF_nnMS (n=3,472), and their drug resistances were evaluated by applying regularized elastic net regression analysis and Fisher’s exact test. The mutations with mutation frequency less than 5% (n=15) are filtered out. Then, by applying a prior knowledge-based selection of the candidate gene-drug pairs, we selected the cancer-related genes (n=18) which were obtained from allOnco database (n=2,125). In addition, previously known drug–gene interactions were filtered out (n=7), which were obtained from CCLE, PubChem database and PubMed literatures.

### Public data and softwares

The following public data and softwares are used in the analysis.

TCGA, https://cancergenome.nih.gov/

CCLE, http://www.broadinstitue.org/ccle

GSEA, http://software.broadinstitute.org/gsea/index.jsp

MSigDB, http://software.broadinstitute.org/gsea/msigdb

allOnco, <http://www.bushmanlab.org/links/genelists>

PubChem, http://pubchem.ncbi.nlm.nih.gov

Tophat, https://ccb.jhu.edu/software/tophat

Cufflinks, <http://cole-trapnell-lab.github.io/cufflinks>

cBioPortal, http://www.cbioportal.org/

### Cells, antibodies, and other reagents

Human lung cancer cells of A549 cells, human liver cancer cells of Huh7, and human skin cancer cells of A375P cells were purchased from Korean Cell Line Bank (KCLB) and cultured in RPMI supplemented with 10% FBS, 100 U/ml penicillin, and 100 µg/ml streptomycin. Anti-EPHB6, anti-phospho-EPHA2 (Ser897), anti-c-Cbl, anti-phospho-SAPK/JNK (T183/Y185), anti-SAPK/JNK, anti-phospo-FAK (Y397), anti-FAK antibody, and Dylight 594 phalloidin were purchased from Cell signaling Biotechnology (Danvers, MA, USA). Anti-phospho-c-Jun (S63) and anti-c-Jun antibodies were from BD Biosciences (San Jose, CA, USA), anti-EPHA2 and anti-β-actin antibodies were from Santa Cruz Biotechnology (Dallas, TX, USA), anti-vinculin antibodies were from Novus Biologicals (Littleton, CO, USA), paclitaxel, SP600125, Y27632, and FAK inhibitor 14 were from Sigma-Aldrich (St. Louis, MO, USA), and ALW-II-41-27 was from APExBIO (Houston, TX, USA).

### Gene expression constructs and lentiviral vector transfection

Lentiviral constructs expressing *CDH11* shRNA and *JUN* shRNA were purchased from Sigma-Aldrich (St. Louis, MO, USA). The *EPHB6*-wild type, *EPHB6*-Q926R, *EPHB6*-del915-917 cDNA constructs were cloned into pCDH-CMV-MCS-EF1-Puro, a lentiviral vector for cDNA expression (System Biosciences, Mountain View, CA, USA). All the lentiviral vectors were transfected into 293TN cells (System Biosciences) with Lipofectamin 3000 transfection reagent (Invitrogen, Waltham, MA, USA). Particles were collected 2 days after the transfection of the lentiviral plasmids and were used to infect cancer cells. Lentivirus-infected cancer cells were puromycin-selected for 1 week.

### RNA-seq profiling

Total RNA was extracted from each sample using the mirVana total RNA extraction kit (Ambion, Austin, TX). The sequencing library for RNA was constructed using TruSeq RNA sample preparation kit (illumina, San Diego, CA) according to manufacturer’s instruction. Sequencing reaction was performed on an illumina NextSeq 500 for paired end reads (2 X 75) with coverage greater than 30 million reads per sample. The raw image data was transformed and stored in FASTQ format. The sequence reads were mapped to the human reference genome (hg38) and RNA abundance was estimated by using Tophat-cufflinks with default parameters, and log2 transformed FPKM values were used.

### *In vivo* experiments

Vector, WT, or Q926R cells (1 X 10^7^ cells /100 µl) and matrigel (Corning, Bedford, MA, USA) 100 ul mixtures (total, 200 ul/head) were injected subcutaneously in right rear dorsal flank region of Balb/c nude mouse. When the tumor volume reached approximately 50 mm^3^, the mice were randomized into two treatment group: Control, 20 mg/kg paclitaxel. Paclitaxel was administered on day 1, 3, and 5 via intraperitoneal injection [[3](#_ENREF_3)]. Tumors were measured using an optical caliper with a 3-day interval and tumor size was calculated using the following formula: length x (width)^2^ x 0.5. All surgical and experimental procedures were approved by the institutional animal care and use committee at the Ajou University, College of Medicine.

### RhoA GTPase activity assay

RhoA activity was measured by using a kit from Cell Biolabs (San Diego, CA, USA). Briefly, cell lysates were incubated with agarose beads coupled to the Rho-binding domain (RBD) of Rhotekin. The amount of bound form Rho A was measured by western blot analysis using an anti-RhoA antibody.

### Cell adhesion assay

Cell adhesion was measure by a colorimetric-based assay (CytoSelect 48-well Cell Adhesion Assay; Cell Biolabs Inc.) according to the manufacturer`s instructions. Briefly, cells were serum starved for 24 hr prior to seeding on collagen type IV-coated adhesion plate at a concentration of 1 x 10^6^ cells/mL in serum-free media. Cells were incubated for 90 min. Non-adherent cells were gently removed by several washes with 1 x PBS, then the adherent cells were fixed by 3.7% formaldehyde and stained by Coomassie Brilliant Blue. The adherent cells were dissolved by an extraction solution, and the absorbance of this solution was measured at 560 nm in a microplate reader.

### *In vitro* drug sensitivity assay

To estimate CAM-DR*, in vitro* drug sensitivity assay was performed in 6-well plates as previously described [[4](#_ENREF_4)]. Cells (1 x 10^3^ cells) were pre-incubated with or without of indicated drugs for 15 min, and then adhered to the plates coated by collagen type IV. After overnight incubation at 37 °C for adhesion, paclitaxel (10 nM) was added and the incubation was continued for 24 hr. After washing the plates twice with serum-free RPMI-1640, cells were grown in complete culture medium for 14 days. The resulting colonies grown in the plates were stained with Coomassie Brilliant Blue, and the visible number of colonies were counted.

### Real-time qPCR

Cells were harvested and total RNAs were isolated using an RNeasy kit (Qiagen, Venlo, Netherlands). The PrimeScript RT kit (Takara, Shiga, Japan) was used to reverse transcribed the mRNA into cDNA. PCR was done using a CFX96 Real Time PCR Detection System (Bio-Rad Laboratories, Hercules, CA, USA) with Ssoadvanced Universal Supermixes (Bio-Rad). Analysis of each sample was performed at least three times for each experiment, and the data in the figures were reported as relative quantitation: average values of 2^-ΔΔCT^±S.D. (standard deviation). The sequences of primers are as follows.

| Gene | 5’ primer | 3’ primer |
| --- | --- | --- |
| *CDH11* | 5`- GCACCATGAGAAGGGCAAGG -3` | 5`- AATGAAGCCTGCCCACAAGC -3` |
| *EPHB6* | 5`- GGAGTGCTACCAGGACAACT -3` | 5`- GTTGCTGAAGGAGCTGGATG-3` |
| *β-actin* | 5`- TGGCACCCAGCACAATGAA -3` | 5`- CTAAGTCATAGTCCGCCTAGAAGCA -3` |

### Immunoprecipitation and Western blots

For immunoprecipitation, 200 µg of proteins were incubated overnight with anti-EPHB6 antibody or anti-EPHA2 antibody, subsequently with protein A/G agarose beads (Santa Cruz Biotechnology) for 1 h. The beads were washed five times from which immunoprecipitates were extracted with 2 Χ SDS sample buffer. For preparing total cell lysates, cells were lysed in high salt lysis buffer [20 mM Tris-HCl [pH 8.0], 1% Triton X-100, 2 mM EDTA, and 1 mM phenylmethylsulfonyl fluoride], incubated on ice for 20 min, and centrifuged for 20 min to remove cell debris. A total of 20 μg of whole-cell lysate was used in SDS-polyacrylamide gel electrophoresis. The proteins were then electro-transferred to a nitrocellulose membrane and incubated overnight with antibodies at 4°C. Then, the membranes were incubated with peroxidase-conjugated secondary antibodies (PIERCE, Rockford, IL, USA) for 1 hr at room temperature, and the signal was detected using an enhanced chemi-luminescence detection kit (PIERCE).

### Confocal microscopy

Cells were grown on Lab-Tek four-well glass chamber slides (NUNC, Rockford, IL, USA). After 24 hr incubation, cells were fixed by methanol-free 4% formaldehyde for 15 min. Following incubation of the primary and secondary antibodies, the cells were treated by DyLight 594 Phalloidin diluted 1:20 in PBS, incubated for 15 min at room temperature, then rinsed three times with PBS. Cell images were collected by a laser scanning confocal microscope LSM710 (Carl Zeiss, Oberkochen, Germany) equipped with argon (488 nm) and krypton (568 nm) lasers, using a Χ 40 water immersion objective lens. Images were processed with ZEN 2009 light edition (Carl Zeiss).

### Cell proliferation, migration, and invasion assay

Cells (5 Χ 10^3^) were split into 96-well plates, and incubated in RPMI containing 5% FBS with the indicated concentration of paclitaxel for 48 hr. Cell viability was measured by WST-1 assay (Roche, Gangnam-gu, Seoul. Korea). Each experiment was performed in four replicates at least three times.

Cell migration and invasion assay were performed in 24-well modified Boyden chamber (8-μm pore size; Coastar; Corning Life Sciences, Lowell, MA). For invasion assay, the transwell filter inserts were coated with collagen type I (Sigma-Aldrich, St. Louis, MO, USA). In the migration assay, 1ⅹ10^5^ cells in 0.2 ml of serum-free DMEM were seeded into the upper chamber insert. The lower chamber was filled with 0.6 ml DMEM/10% fetal bovine serum. After 6 hrs, cells at the membrane undersurface were fixed, stained, and counted at ⅹ200 magnification for four microscopic fields.
