# Supplementary material for: EPHB6 mutation induces cell adhesion-mediated paclitaxel resistance via EPHA2 and CDH11 expression
Source: Exp Mol Med. 2019 Jun 3;51(6):61. doi: 10.1038/s12276-019-0261-z (PMC6547695; doi:10.1038/s12276-019-0261-z)
Supplement: Supplementary file 2 — Supplementary Figure [file 12276_2019_261_MOESM2_ESM.pdf]

Supplementary Figures

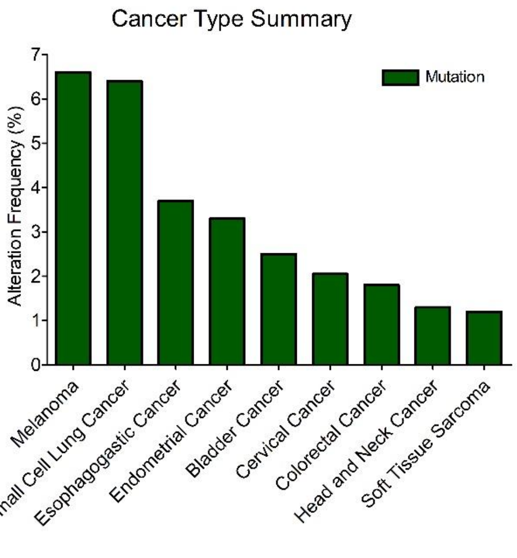

Supplementary Figure 1. Mutation frequencies of EPHB6 across cancer types

Mutation frequencies of *EPHB6* are shown. Of the 32 cancer types of provisional data, the 9 cancer types are selected which have more than 100 samples and their mutation frequencies greater than 1%. The plot is generated from cBioPortal database.

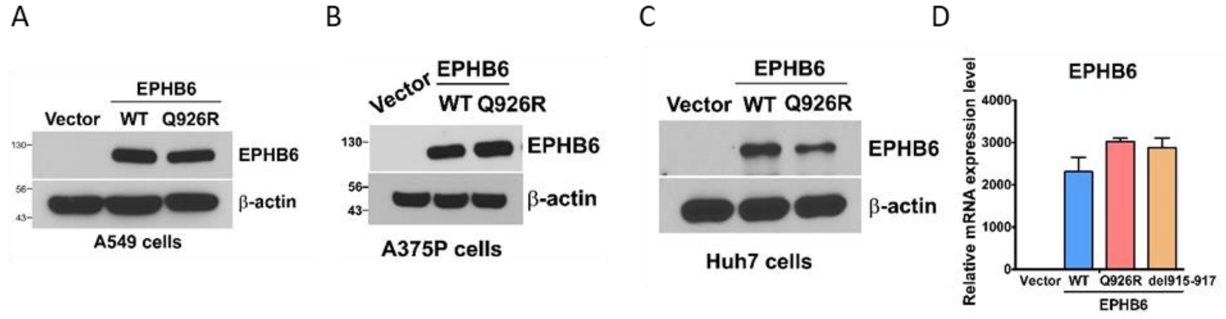

Supplementary Figure 2. Expression of the wild-type or mutant types of *EPHB6*

(**A-C**) A549 (**A**), A375P (**B**), or Huh7 cells (**C**) stably expressing empty vector (Vector), *EPHB6*-wild type

(WT), or *EPHB6*-Q926R (Q926R) are western blotted with indicated antibodies. (**D**) *EPHB6* mRNA expression levels are measured by qRT-PCR in the indicated cells.

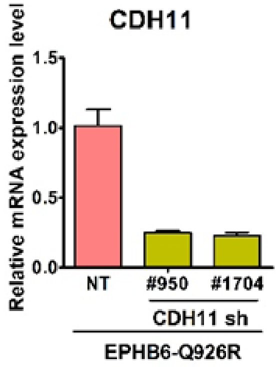

Supplementary Figure 3. Expression of CDH11 in EPHB6-Q926R cells.

EPHB6-Q926R cells stably expressing non-targeting (NT) shRNA, *CDH11* shRNA (#950 and #1704) are established. qRT-PCR for CDH11 is performed. Values are means ±SEM of three independent experiments

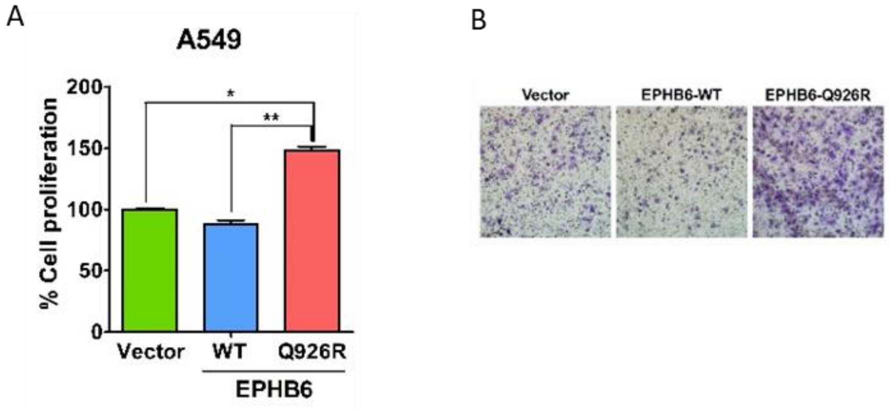

Supplementary Figure 4. The effect of *EPHB6* mutation on cell proliferation and migration.

(A) The proliferation of the indicated cells is measured by WST-1 assay after 72 hr incubation. *P < 0.05 and **P < 0.01. **(B)** Cell migration activities of the Vector, WT, or Q926R cells are measured by

transwell assay systems. After 4 hr incubation, the invaded cells are fixed and stained using light microscopy (magnification, X 200).
