# Supplementary material for: EPHB6 mutation induces cell adhesion-mediated paclitaxel resistance via EPHA2 and CDH11 expression
Source: Exp Mol Med. 2019 Jun 3;51(6):61. doi: 10.1038/s12276-019-0261-z (PMC6547695; doi:10.1038/s12276-019-0261-z)
Supplement: Supplementary file 3 — Supplementary Tables [file 12276_2019_261_MOESM3_ESM.docx]

Supplementary Tables

## Supplementary Table 1. List of drug resistance associated mutations identified from CCLE data analysis

| Feature Type | Drug | Predicted gene | CCLE/PubChem target | Frequency | Odds Ratio/EN coefficients | Lower 95% CI | Upper 95% CI | P-value/1-r | Analysis type |
| --- | --- | --- | --- | --- | --- | --- | --- | --- | --- |
| mutLOF_nnMS | Paclitaxel | EPHB6 | TUBB1/Microtublues | 16 | 0.20 | 0.04 | 0.76 | 0.009 | Fisher |
| mutLOF_nnMS | Panobinostat | TNN | HDAC | 21 | 0.23 | 0.05 | 0.72 | 0.006 | Fisher |
| mutLOF | Irinotecan | CLTCL1 | TOP1 | 173 | -1.61 | -1.74 | -1.48 | 0.05 | ElasticNet |
| mutLOF | Topotecan | CLTCL1 | TOP1 | 266 | -1.87 | -2.04 | -1.69 | 0.01 | ElasticNet |

## Supplementary Table 2. List of *EPHB6* mutations associated with paclitaxel resistance

| Position | Ref | Alt | Variant  classification | cDNA  change | Codon  change | Protein  change | Cell Name | Tissue | IC50  (uM) |
| --- | --- | --- | --- | --- | --- | --- | --- | --- | --- |
| chr7:142568136 | A | G | Missense  mutation | c.2777A>G | c.(2776-2778)CAG>CGG | p.Q926R | EBC-1 | LUNG | 8 |
| chr7:142566326 | B | A | Nonsense  mutation | c.2115G>A | c.(2113-2115)TGG>TGA | p.W705* | NCI-H1373 | LUNG | 8 |
| chr7:142561954 | C | A | Nonsense  mutation | c.396C>A | c.(394-396)TAC>TAA | p.Y132* | SK-MEL-5 | SKIN | 8 |
| chr7:142562408 | C | A | Missense  mutation | c.850C>A | c.(850-852)CTG>ATG | p.L284M | SF126 | CENTRAL  NERVOUS  SYSTEM | 0.068713717 |
| chr7:142561960 | G | T | Missense  mutation | c.402G>T | c.(400-402)CAG>CAT | p.Q134H | T3M-10 | LUNG | 0.062078975 |
| chr7:142562228 | G | A | Missense  mutation | c.670G>A | c.(670-672)GCT>ACT | p.A224T | IGROV1 | OVARY | 0.027501697 |
| chr7:142566454 | C | T | Missense  mutation | c.2243C>T | c.(2242-2244)ACG>ATG | p.T748M | SK-MEL-2 | SKIN | 0.026818315 |
| chr7:142567588 | C | T | Missense  mutation | c.2476C>T | c.(2476-2478)CGC>TGC | p.R826C | MeWo | SKIN | 0.023242673 |
| chr7:142561412 | G | A | Missense  mutation | c.124G>A | c.(124-126)GAG>AAG | p.E42K | NCI-H1651 | LUNG | 0.022404801 |
| chr7:142566258 | G | T | Nonsense  mutation | c.2047G>T | c.(2047-2049)GAA>TAA | p.E683* | SW 900 | LUNG | 0.0188492 |
| chr7:142563299 | A | C | Missense  mutation | c.1016A>C | c.(1015-1017)CAC>CCC | p.H339P | LN-229 | CENTRAL  NERVOUS  SYSTEM | 0.015199102 |
| chr7:142562030 | A | G | Missense  mutation | c.472A>G | c.(472-474)ATT>GTT | p.I158V | NCI-H1339 | LUNG | 0.014453353 |
| chr7:142564781 | G | T | Missense  mutation | c.1705G>T | c.(1705-1707)GGC>TGC | p.G569C | NCI-H1339 | LUNG | 0.014453353 |
| chr7:142564775 | G | T | Missense  mutation | c.1699G>T | c.(1699-1701)GGC>TGC | p.G567C | NCI-H441 | LUNG | 0.013821052 |
| chr7:142566113 | C | A | Missense  mutation | c.2033C>A | c.(2032-2034)ACA>AAA | p.T678K | NCI-H441 | LUNG | 0.013821052 |

## Supplementary Table 3. List of the differentially up- and down-regulated genes between the EPHB6 (Q926R) and EPHB6 (WT) cells

| Symbol | Fold change |
| --- | --- |
| Up-regulated genes | |
| CDH11 | 2.462 |
| MAL2 | 2.196 |
| SLC6A14 | 2.148 |
| ESRP1 | 1.773 |
| TFF1 | 1.769 |
| KRT19 | 1.632 |
| CA2 | 1.531 |
| CST1 | 1.499 |
| CES1 | 1.479 |
| CEMIP | 1.439 |
| AQP3 | 1.411 |
| PCDH7 | 1.381 |
| CELF2 | 1.369 |
| TLL1 | 1.353 |
| MUC5B | 1.347 |
| FAAH2 | 1.281 |
| SNCA | 1.269 |
| FXYD3 | 1.234 |
| MAPK4 | 1.228 |
| TNFSF15 | 1.218 |
| SERPINB7 | 1.174 |
| GALNT3 | 1.165 |
| ST6GALNAC1 | 1.141 |
| UNC5D | 1.118 |
| ICA1 | 1.098 |
| CST2 | 1.072 |
| MARK1 | 1.026 |
| LAMA4 | 1.024 |
| COL4A5 | 1.019 |
| TSPAN1 | 0.991 |
| COL4A6 | 0.968 |
| TGM2 | 0.967 |
| EPCAM | 0.948 |
| ADAP1 | 0.936 |
| EFNA1 | 0.895 |
| CST4 | 0.893 |
| ITGAM | 0.889 |
| SVIL | 0.887 |
| MILR1 | 0.876 |
| MAP4K1 | 0.872 |
| USH1C | 0.845 |
| SCEL | 0.843 |
| PIP | 0.838 |
| EVA1A | 0.830 |
| KRT6A | 0.825 |
| HIST1H2BJ | 0.811 |
| RGS2 | 0.809 |
| TSPAN8 | 0.796 |
| DCLK1 | 0.790 |
| CCL26 | 0.784 |
| SP8 | 0.781 |
| IGFL4 | 0.765 |
| FGFBP1 | 0.756 |
| TRIM29 | 0.755 |
| ARMC3 | 0.741 |
| ATP6V1G2-DDX39B | 0.737 |
| SMIM22 | 0.737 |
| PASK | 0.734 |
| DMKN | 0.726 |
| S100A9 | 0.725 |
| THEM5 | 0.724 |
| CEACAM6 | 0.701 |
| CKMT1B | 0.701 |
| STYK1 | 0.697 |
| RNF128 | 0.693 |
| OVOL2 | 0.692 |
| PKIB | 0.674 |
| GABRA2 | 0.666 |
| CEACAM1 | 0.664 |
| TRPV6 | 0.661 |
| TMCO4 | 0.660 |
| TNS4 | 0.655 |
| MOB3B | 0.654 |
| GDA | 0.652 |
| MGLL | 0.650 |
| LSR | 0.649 |
| NR4A2 | 0.645 |
| ZNF596 | 0.644 |
| TC2N | 0.641 |
| LCE5A | 0.641 |
| PDZK1 | 0.638 |
| CBLB | 0.637 |
| STARD4 | 0.635 |
| CA9 | 0.634 |
| HPD | 0.633 |
| KRT20 | 0.633 |
| PCLO | 0.631 |
| CKMT1A | 0.631 |
| SULT1A1 | 0.631 |
| IQGAP2 | 0.631 |
| FAM83E | 0.630 |
| P2RX5 | 0.624 |
| APOL2 | 0.624 |
| BSPRY | 0.623 |
| SLC6A15 | 0.619 |
| GAB1 | 0.617 |
| BMPER | 0.615 |
| DACT2 | 0.611 |
| ZNF223 | 0.611 |
| MARVELD3 | 0.608 |
| NAV1 | 0.599 |
| PLAC8 | 0.599 |
| ZNF235 | 0.596 |
| KHDRBS3 | 0.596 |
| CDK7 | 0.593 |
| IAPP | 0.592 |
| EPB41L3 | 0.587 |
| CNTNAP2 | 0.586 |
| TRIM31 | 0.578 |
| SRGN | 0.578 |
| SLITRK6 | 0.576 |
| ADGRL3 | 0.574 |
| GPR157 | 0.572 |
| FIGN | 0.569 |
| CFAP57 | 0.569 |
| CCDC30 | 0.568 |
| DDIT4L | 0.568 |
| MFAP5 | 0.565 |
| COL17A1 | 0.564 |
| CEACAM5 | 0.563 |
| SOX21 | 0.562 |
| TTC28 | 0.558 |
| FCGBP | 0.556 |
| NTN1 | 0.556 |
| NIPAL1 | 0.554 |
| S100A14 | 0.552 |
| SLC26A3 | 0.552 |
| CXCL8 | 0.551 |
| NMRK1 | 0.549 |
| LUM | 0.548 |
| EIF5A2 | 0.548 |
| ALDH1A3 | 0.547 |
| SMOX | 0.545 |
| CPS1 | 0.545 |
| ZNF425 | 0.541 |
| HERC3 | 0.540 |
| SGK3 | 0.539 |
| TECPR1 | 0.539 |
| SPATA25 | 0.537 |
| RAB17 | 0.536 |
| HMGA2 | 0.535 |
| GPNMB | 0.532 |
| STC1 | 0.530 |
| SPOCK3 | 0.529 |
| KLK5 | 0.529 |
| IL1R2 | 0.526 |
| SYK | 0.525 |
| PCSK9 | 0.523 |
| URGCP-MRPS24 | 0.520 |
| MYLPF | 0.520 |
| CLDN3 | 0.519 |
| CD55 | 0.519 |
| OLR1 | 0.517 |
| OR51E1 | 0.516 |
| NDRG2 | 0.513 |
| MUC1 | 0.512 |
| ZRANB3 | 0.511 |
| PLA2G12A | 0.511 |
| FRS2 | 0.511 |
| RIOK2 | 0.510 |
| PPARGC1A | 0.510 |
| TANGO2 | 0.509 |
| TTC39C | 0.509 |
| FSTL4 | 0.509 |
| RALGAPA2 | 0.508 |
| BCL2L15 | 0.507 |
| SYNGR4 | 0.505 |
| STK38L | 0.505 |
| HAPLN1 | 0.503 |
| STON2 | 0.502 |
| SAP25 | 0.501 |
| Down-regulated gene | |
| RIMS1 | -0.964 |
| PYURF | -0.926 |
| BRICD5 | -0.923 |
| RPS4Y1 | -0.908 |
| CTNND2 | -0.882 |
| DDX3Y | -0.875 |
| PSORS1C1 | -0.838 |
| KDM5D | -0.822 |
| DEFB1 | -0.820 |
| HIST1H4E | -0.797 |
| DPYSL5 | -0.770 |
| TLE2 | -0.763 |
| AGBL3 | -0.762 |
| CT45A10 | -0.757 |
| HIST1H2AI | -0.746 |
| DEPDC5 | -0.741 |
| NDRG4 | -0.727 |
| SSBP2 | -0.722 |
| EIF1AY | -0.717 |
| FXYD6 | -0.692 |
| FAM19A5 | -0.692 |
| SDC2 | -0.682 |
| HOXB2 | -0.680 |
| FAM27C | -0.680 |
| GLI2 | -0.680 |
| RHOD | -0.676 |
| LGALS2 | -0.657 |
| GRIP1 | -0.630 |
| CDKL5 | -0.625 |
| NPL | -0.622 |
| NLGN2 | -0.622 |
| SAGE1 | -0.620 |
| ADAMTS10 | -0.611 |
| PDE8B | -0.603 |
| GIP | -0.591 |
| KRT4 | -0.582 |
| CT45A9 | -0.581 |
| AIFM3 | -0.580 |
| COL11A1 | -0.579 |
| MYCBPAP | -0.579 |
| ITGBL1 | -0.577 |
| CLMP | -0.574 |
| NDUFA4L2 | -0.572 |
| NEIL1 | -0.568 |
| ISYNA1 | -0.566 |
| GAFA3 | -0.565 |
| CIART | -0.564 |
| TNS2 | -0.562 |
| CDH23 | -0.560 |
| ABCC8 | -0.560 |
| SLC22A17 | -0.558 |
| ABHD14A-ACY1 | -0.557 |
| RBFOX3 | -0.556 |
| EXOC3L4 | -0.554 |
| KCTD17 | -0.553 |
| HIST1H1E | -0.552 |
| ZNF487 | -0.548 |
| SYNC | -0.547 |
| TDRP | -0.547 |
| TMEM189-UBE2V1 | -0.547 |
| CTXN1 | -0.543 |
| ANKRD13B | -0.543 |
| ARHGAP22 | -0.542 |
| PIH1D2 | -0.540 |
| SPANXC | -0.539 |
| DSC3 | -0.539 |
| HCG27 | -0.539 |
| DCAF17 | -0.538 |
| MDH1B | -0.537 |
| SBK2 | -0.536 |
| CCDC69 | -0.532 |
| GSTM2 | -0.531 |
| PLSCR4 | -0.530 |
| FXYD2 | -0.529 |
| PIBF1 | -0.528 |
| DMC1 | -0.528 |
| TTLL3 | -0.528 |
| SORCS2 | -0.527 |
| PIP5KL1 | -0.526 |
| PLD5 | -0.522 |
| ZNF628 | -0.522 |
| PPP2R5B | -0.519 |
| PRR5L | -0.516 |
| IGSF1 | -0.515 |
| MAPK10 | -0.515 |
| UGT1A3 | -0.514 |
| USP9Y | -0.513 |
| NMUR2 | -0.513 |
| ADH4 | -0.511 |
| CHTF18 | -0.509 |
| FKSG48 | -0.508 |
| PBX4 | -0.507 |
| SYT1 | -0.506 |
| STAC3 | -0.504 |
| PLPPR3 | -0.504 |
| CT45A8 | -0.502 |
| LCOR | -0.501 |
| TRPS1 | -0.501 |

## Supplementary Table 4. Functional enrichment for the differentially up-regulated genes (EPHB6_MT) between the EPHB6 (Q926R) and EPHB6 (WT) cells

| **EPHB6_MT** | |  |  |
| --- | --- | --- | --- |
| **Annotation Cluster 1** | **Enrichment Score: 2.6970210102753014** |  |  |
| **Category** | **Term** | **Count** | **P value** |
| GOTERM_BP_FAT | GO:0051674~localization of cell | 31 | 6.04E-06 |
| GOTERM_BP_FAT | GO:0040012~regulation of locomotion | 20 | 1.15E-04 |
| GOTERM_BP_FAT | GO:0016477~cell migration | 26 | 1.17E-04 |
|  |  |  |  |
| **Annotation Cluster 2** | **Enrichment Score: 1.847725383777281** |  |  |
| **Category** | **Term** | **Count** | **P value** |
| GOTERM_BP_FAT | GO:0018209~peptidyl-serine modification | 9 | 3.21E-03 |
| GOTERM_BP_FAT | GO:0018105~peptidyl-serine phosphorylation | 7 | 2.92E-02 |
| GOTERM_BP_FAT | GO:0018193~peptidyl-amino acid modification | 19 | 3.04E-02 |
|  |  |  |  |
| **Annotation Cluster 3** | **Enrichment Score: 1.6737973681011604** |  |  |
| **Category** | **Term** | **Count** | **P value** |
| GOTERM_BP_FAT | GO:0008283~cell proliferation | 29 | 8.33E-03 |
| GOTERM_BP_FAT | GO:0042127~regulation of cell proliferation | 24 | 1.96E-02 |
| GOTERM_BP_FAT | GO:0008284~positive regulation of cell proliferation | 14 | 5.81E-02 |
